# Mitogenic growth signalling, DNA replication licensing, and survival are linked in prostate cancer

**DOI:** 10.1038/sj.bjc.6603718

**Published:** 2007-04-03

**Authors:** T J Dudderidge, S R McCracken, M Loddo, T R Fanshawe, J D Kelly, D E Neal, H Y Leung, G H Williams, K Stoeber

**Affiliations:** 1 Department of Pathology and Royal Free and University College Medical School, University College London, Rockefeller Building, University Street, London, WC1E 6JJ, UK; 2 Northern Institute for Cancer Research, University of Newcastle, Paul O'Gorman Building, Medical School, Framlington Place, Newcastle upon Tyne, NE2 4HH, UK; 3 Department of Public Health and Primary Care, Centre for Applied Medical Statistics, Institute of Public Health,University of Cambridge, Forvie Site, Robinson Way, Cambridge, CB2 2SR, UK; 4 Department of Oncology and Hutchison MRC Research Centre, University of Cambridge, Hills Road, Cambridge, CB2 2XZ, UK; 5 Wolfson Institute for Biomedical Research, University College London, The Cruciform Building, Gower Street, London, WC1E 6BT, UK

**Keywords:** MEK5/ERK5, Mcm2, geminin, DNA replication licensing, prognosis, prostate cancer

## Abstract

Activation of mitogen/extracellular-signal-regulated kinase kinase 5/extracellular signal-regulated kinase-5 (MEK5/ERK5) growth signalling is coupled to increased cell proliferation in prostate cancer (PCa). Dysregulation of the DNA replication licensing pathway, a critical step in growth control downstream of transduction signalling pathways, is associated with development of PCa. In this study we have investigated linkages between the MEK5/ERK5 pathway and DNA replication licensing during prostate carcinogenesis. The effects of increased MEK5/ERK5 signalling on the expression of replication licensing factors Mcm2 and geminin and the proliferation marker Ki67 were studied in an ecdysone-inducible system expressing a constitutively activated mutant of MEK5 in EcR293 cells and in stable ERK5 over-expressing PC3 clones. In parallel, expression of these biomarkers in PCa biopsy specimens (*n*=58) was studied and compared to clinicopathological parameters. In both *in vitro* systems induction of MEK5 expression resulted in increased levels of phosphorylated ERK5 and Mcm2, geminin and Ki67 proteins. In PCa specimens average Mcm2 expression was greater than Ki67 and geminin expression (median labelling index (LI) 36.7, 18.1, and 3.4% respectively), consistent with their differential expression according to growth status (*P*<0.0001). Mcm2, geminin and Ki67 expression were significantly associated with Gleason grade (*P*=0.0002, *P*=0.0003, *P*=0.004); however there was no link with T or M stage. There was a significant relationship between increasing ERK5 expression and increasing Mcm2 (*P*=0.003) and Ki67 (*P*=0.009) expression, with non-significant trends seen with increasing MEK5 expression. There were significant associations between Gleason grade and the number of cells traversing G1 phase (Ki67_LI_-geminin_LI_; (*P*=0.001)), with high ERK5 levels associated with both an increase in replication licensed but non-cycling cells (Mcm2_LI_-Ki67_LI_; (*P*=0.01)) and accelerated cell cycle progression (geminin_LI_/Ki67_LI_; (*P*= 0.005)), all indicative of a shift towards increasing proliferative potential. While Mcm2 and Ki67 were both prognostic factors on univariate analysis, only Mcm2 remained an independent prognostic marker on multivariate analysis. Taken together, our data show that induction of MEK5/ERK5 signalling is linked to activation of the DNA replication licensing pathway in PCa, and that the strong prognostic value of MCM proteins may result from their function as relay stations coupling growth regulatory pathways to genome duplication.

Prostate cancer (PCa) is the commonest cancer affecting men in the UK, with 30 140 new cases per year representing 22% of new male cancers. The 5-year relative age standardized survival for patients diagnosed with PCa is 65%, accounting for 9940 deaths per year and making PCa the second leading cause of cancer death (Cancer Research UK, 2005). PCa has a broad spectrum of pathological behaviour. Many patients with PCa will die of other causes due to the indolent nature of their disease, while others will progress and die of metastatic disease despite radical therapy. Treatment selection is based on patient's age and health status, as well as prognostic factors such as Gleason grade, TNM stage and serum prostate specific antigen (PSA). Histological Gleason grade currently gives the most accurate assessment of growth status of PCa; however, it is prone to inter- and intra-observer error (Gleason, 1992). As for many cancers, improved prognostic markers for clinical application are urgently sought to aid treatment selection and to identify high-risk patients.

The mitogen/extracellular-signal-regulated kinase kinase 5/extracellular signal-regulated kinase-5 (MEK5/ERK5) pathway has been implicated in the regulation of cell proliferation. Mitogen-activated protein (MAP) kinase kinases (MEKs/MAPKKs) represent a family of protein kinases upstream of MAP kinases that play a pivotal role in regulation of cell proliferation and apoptosis. MEK5 is the most recently identified MAPKK and encodes a 444-amino-acid protein with an overall 40% homology to other MEK proteins (English *et al*, 1995; Zhou *et al*, 1995). MAPKK specifically activates ERK5, also known as Big MAP kinase 1, which is almost twice the size of other MAPKs (Zhou *et al*, 1995). The activities of several transcriptional factors have been shown to be regulated by ERK5, including MEF2, c-Fos and Fra1, Sap-1, c-Myc and NF-*κ*B (Kato *et al*, 1997; English *et al*, 1998; Pearson *et al*, 2001; Barsyte-Lovejoy *et al*, 2002; Terasawa *et al*, 2003). We have previously demonstrated that MEK5 overexpression is associated with metastatic PCa, and that it is able to induce proliferation, motility and invasion in PCa cells. Moreover increased MEK5 and ERK5 expression correlates with presence of bony metastases and less favourable disease-specific survival (Mehta *et al*, 2003). These independent markers of prognosis are currently being tested as stratification tools for identification of PCa with an aggressive phenotype.

The initiation of DNA replication is a critical step in growth control that lies downstream of the RAS-RAF-MEK-MAP kinase signal transduction pathway. It is dependent on the assembly onto chromatin of pre-replicative complexes (pre-RCs) containing Orc1–6, Cdc6, Cdt1 and minichromosome maintenance (Mcm) 2–7 proteins during G1 phase, thereby rendering replication origins ‘licensed’ for one round of DNA synthesis during S phase. At the G1-S transition pre-RCs are activated by cyclin-dependent kinases (CDKs) and Cdc7/ASK kinase, resulting in recruitment of additional initiation factors and DNA polymerase *α*-primase to the origin, unwinding of the DNA helix, and initiation of DNA synthesis (Machida *et al*, 2005). DNA replication initiation is tightly coupled to removal of the license. This step is critical as origins must fire once-and-only-once in each cell cycle to ensure genomic stability. To avoid re-licensing, mammalian cells have adopted a number of mechanisms. These include inactivation of replication licensing factors (RLFs) during S, G2 and M phase (a process controlled by CDK activity), regulated proteolysis, and changes in gene expression (Machida *et al*, 2005). Arguably the most important mechanism of preventing re-replication is the expression of a repressor of origin licensing, known as geminin, which acts by competitively binding Cdt1, thereby blocking MCM loading and hence pre-RC re-assembly onto chromatin (Blow and Dutta, 2005). Geminin expression is tightly restricted to S, G2 and M phase of the cell cycle, in keeping with its function of preventing re-replication (Wharton *et al*, 2004). The constituents of the pre-RC can be regarded as relay stations coupling growth regulatory pathways with DNA replication (Williams and Stoeber, 1999). We and others have demonstrated in a range of different tumour types that dysregulation of the MCM proteins is an early event in tumourigenesis and have exploited these biomarkers in primary diagnosis, tumour surveillance and prognosis (Williams *et al*, 1998, 2004; Stoeber *et al*, 1999, 2002; Wharton *et al*, 2001, 2004; Davies *et al*, 2002; Going *et al*, 2002; Gonzalez *et al*, 2003; Kruger *et al*, 2003; Padmanabhan *et al*, 2004; Dudderidge *et al*, 2005; Korkolopoulou *et al*, 2005; Shetty *et al*, 2005). These studies have established that the superior sensitivity of MCM proteins over the standard proliferation marker Ki67 resides in the fact that these biomarkers identify not only cycling cells but also non-cycling cells with proliferative potential (Stoeber *et al*, 2001). Moreover, we have shown that multiparameter analysis of tumour cell populations using Mcm2–7, geminin and Ki67 biomarkers allows a detailed analysis of cell cycle kinetics in biopsy material (Wharton *et al*, 2004; Obermann *et al*, 2005).

Here we have investigated the regulation of growth control in PCa by investigating linkages between MEK5/ERK5 growth signalling and the downstream DNA replication (or origin) licensing pathway (RLP). The MAPK-NF*κ*B pathway is a potent regulator of cell proliferation through transcriptional control of cyclin D1 (Guttridge *et al*, 1999; Hinz *et al*, 1999), and several of the genes encoding RLFs appear to be E2F regulated and their promoter activities growth-dependant (Blow and Hodgson, 2002). First, we used an ecdysone-inducible *in vitro* system expressing a constitutively activated mutant of MEK5 in EcR293 cells in addition to stable ERK5 overexpressing PC3 clones to determine the effects of increased MEK5/ERK5 signalling on the RLP *in vitro*. Second, we performed protein expression profiling for MEK5, ERK5, Mcm2, geminin and Ki67 on a series of PCa specimens to investigate the effects of MEK5/ERK5 signalling on origin licensing and cell cycle kinetics *in vivo*.

## MATERIALS AND METHODS

### Antibodies

Mouse anti-human Mcm2 monoclonal antibody (clone 46) was obtained from BD Transduction Laboratories (Lexington, KY, USA). Mouse anti-human Ki67 monoclonal antibody (clone MIB-1) was obtained from DAKO (Glostrup, Denmark). Affinity-purified rabbit polyclonal antibody against full-length *hs*geminin protein was generated as described (Wharton *et al*, 2004). Anti-MEK5 polyclonal antibody was obtained from Stressgen Biotechnologies (San Diego, CA, USA), anti-ERK5 polyclonal and anti-HA monoclonal antibody from Santa Cruz Biotechnology (Santa Cruz, CA, USA), and anti-*α*-tubulin monoclonal antibody from Sigma-Aldrich (Gillingham, Dorset, UK). Anti-ERK5 polyclonal antibody for use in immunohistochemistry was a kind gift from Professor P Cohen (University of Dundee, UK) (Mody *et al*, 2001).

### Plasmid construction and generation of stable clones

An ecdysone-inducible system expressing a constitutively activated mutant of MEK5 in EcR293 cells was used to determine the effects of increased MEK5/ERK5 signalling on the DNA RLP. Stable clone EcRD No. 16 has previously been shown to have negligible background and high Ponasterone A (PonA)-induced MEK5D expression (Mehta *et al*, 2003). Following induction of MEK5 expression with 5 *μ*M PonA for 24 h, whole cell lysates were subjected to SDS–PAGE and immunoblot analysis. In addition to the ecdysone-inducible system, an ERK5 over-expressing PCa stable clone was generated. ERK5 was amplified from a full-length clone (Bouras *et al*, 2001) using oligonucleotides MP405 (GGATCCGCCGAGCCTCTGAAGGAGGAAG) and MP1310 (GCGGATCCTCAGGGGTCCTGGAGGTCAGG) with GC Rich PCR System (Roche, Lewes, East Sussex, UK). The resulting fragment was cloned into pCR2.1 (Invitrogen, Paisley, UK), sequenced, then subcloned into EGFP-C1 (Clontech, Palo Alto, CA, USA) to form EGFP-C1 ERK5. PC3 cells overexpressing ERK5 were generated by transfection with EGFP-C1 ERK5 using Superfect® Transfection Reagent (Qiagen, Crawley, UK) according to the manufacturer's recommendations. Following selection with geneticin (G418, 1 mg ml^−1^; Sigma-Aldrich, Gillingham, Dorset, UK), colonies were left to form for 2–3 weeks. Control stable cell lines were similarly established with the vector (EGFP-C1) alone. Individual colonies were subcultured and screened for ERK5 expression by immunoblotting. Presence and subcellular localisation of ERK5 were also determined by fluorescent microscopy.

### Immunoblotting

Cells were lysed directly on plates with 6 × SDS sample buffer containing 10% *β*-mercaptoethanol. Samples were denatured and proteins separated by SDS–PAGE, followed by transfer to nitrocellulose. Antibodies were used at the following dilutions: anti-HA, 1 : 500; anti-ERK5, 1 : 1000; Mcm2, 1 : 3000; Ki67, 1 : 2500; geminin, 1 : 1000; and anti-*α* tubulin, 1 : 2000. Horseradish peroxidase-conjugated secondary antibodies were applied (1 : 500) and detected using an enhanced chemiluminescence detection kit (ECL, Amersham, Bucks, UK).

### Immunofluorescence and confocal microscopy

PC3 cells stably overexpressing ERK5 (PC3-ERK5) and PC3 cells overexpressing the empty vector alone (PC3-EmptyVector) were seeded onto sterile coverslips in 6-well plates (Corning Incorporated, Corning Life Sciences, One The Valley Centre, Gordon Road, High Wycombe, Bucks, UK) at densities of 3 × 10^4^ cells/well. After 24 h, cells were washed and incubated in Basal Media overnight. Cells were stimulated with EGF for 30 min and immediately fixed with 100% methanol at −20°C for 30 min. To prevent non-specific staining, fixed cells were first blocked in 10% natural rabbit serum (DakoCytomation) for 30 min at RT. Blocking solution was subsequently removed and primary antibodies were added at the following dilutions: anti-ERK5, 1 : 100; anti-Mcm2, 1 : 200. Coverslips were incubated overnight in a humidified atmosphere at 4°C. Cells were washed (× 3) and incubated with rabbit anti-goat secondary antibody (1 : 250, DakoCytomation) and rabbit anti-mouse secondary antibody (1 : 250, DakoCytomation) for 1 h at RT in the dark. Vectashield with 4,6-diamidino-2-phenylindole (DAPI), for nuclear counterstain, was used to mount slides. Images of fixed cells were acquired with a Leica TCS SP2 UV laser-scanning microscope using a × 63 oil immersion lens (1.32 NA DIC). A series of 1 *μ*m vertical optical sections through the entire thickness of the cells was used to produce a Z-series and all images were analysed using LCS 2.00.585 software. Controls, lacking primary antibody, failed to show any staining.

### Human prostate tissue samples

Ethical approval for tissue studies was obtained from the research ethics committee of the Freeman Hospital, Newcastle-upon-Tyne, UK. Consent for the use of surplus tissue obtained at the time of diagnosis was gained from patients undergoing transurethral resection of the prostate for bladder outflow obstruction. Diagnosis of PCa was made by histopathological examination. This cohort of patients was managed by surveillance and subsequent androgen suppression. Formalin-fixed and paraffin wax-embedded tissue blocks containing greater than 75% tumour involvement were selected for analysis, and tissue sections were cut at 4 *μ*m thickness. The cohort included 58 PCa patients with a median age of 75 years (range 55–88). Included in the series were Gleason grades from 6 to 10 (Gleason 6: *n*=9 (16%), Gleason 7: *n*=7 (12%), Gleason 8: *n*=25 (43%), Gleason 9: *n*=12 (21%), Gleason 10: *n*=5 (9%)) and a range of clinical tumour (T) stages as determined by rectal examination (T1: *n*=13 (22%), T2: *n*=15 (26%), T3: *n*=17 (29%), T4: *n*=5 (9%)). Staging data were missing in eight (14%) patients. Nearly all patients (56/58) had a radionucleide bone scan at the time of diagnosis. The group included 28 (48%) patients with and 28 (48%) patients without evidence of bony metastasis. It should be noted that the patients in this cohort were recruited in the pre-PSA era, and thus PSA data were not available. At the time of our study, 45 (78%) patients had died and eight (14%) were alive. Survival times for five patients were unknown, and these patients were excluded from the survival analysis. In the survivors the follow-up ranged from 2 to 142 months, with a mean of 71.6 months (s.d.=55.4).

### Immunohistochemical analysis

#### Mcm2, geminin and Ki67

Immunohistochemical staining for Mcm2, geminin and Ki67 was performed as described (Dudderidge *et al*, 2005). Incubation without primary antibody was used as a negative control, and colonic epithelial sections were used as positive controls. To determine the labelling index (LI) in each tumour, slides were evaluated at low power (× 100) to identify regions of tumour with the most intense degree of staining. From selected areas, three to five fields at × 400 magnification were captured with a charged coupled device camera and analySIS software (SIS, Münster, Germany). Images were subsequently printed for analysis. Quantitative analysis was undertaken with the observer unaware of the clinicopathologic variables. Cells were identified as positive if there was any nuclear staining present, and any stromal or inflammatory cells on the field were excluded. A mean of 893 nuclei were counted for each case. The LI was calculated by dividing the number of positive cells by the total number of cells counted. Reassessment of 10 randomly selected cases by an independent observer showed high inter-observer agreement.

#### MEK5 and ERK5

Immunohistochemical staining for MEK5 was performed as described (Mehta *et al*, 2003). For ERK5 protein expression analysis, paraffin wax-embedded serial sections (4 *μ*m) were incubated overnight at 4°C with anti-ERK5 goat polyclonal antibody at 1 : 200 dilution and immunohistochemical analysis was performed as described (Gnanapragasam *et al*, 2003). The staining was developed with diaminobenzidine tetrahydrochloride (DAB; Sigma, UK), with haematoxylin used as counterstain. Heart muscle was used as a positive control; no primary antibody as a negative control. Levels of MEK5 and ERK5 immunoreactivity were graded as absent (0), weak (1), moderate (2), or strong (3) by two independent observers with high inter-observer agreement.

### Statistical analysis

The association between all-cause mortality and marker expression was assessed using Kaplan–Meier curves and the log-rank test, using the ‘minimum *P*-value approach’ with adjusted *P*-values and a significance level of 0.05 (Faraggi and Simon, 1996). A multivariate Cox proportional hazards model was fitted using a backwards stepwise procedure to investigate trends between marker expression and the standard prognostic variables Gleason grade, T stage, presence of bony metastases, and survival. Statistical significance of trends between markers and standard prognostic variables was assessed using the Mann–Whitney *U* and Jonckheere–Terpstra tests. In this secondary analysis, *P*-values of less than 0.01 were regarded as statistically significant. The Wilcoxon signed-rank test was used to compare overall labelling indices of Mcm2, geminin and Ki67. Analysis was performed using SPSS 12.0 for Windows (SPSS Inc., Chicago, IL, USA) and R version 2.0.

## RESULTS

### Increased MEK5/ERK5 signalling enhances Mcm2, geminin and Ki67 expression

**Figure 1:**
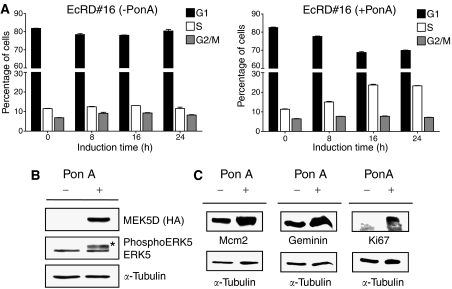
(**A**) FACS analysis. EcRD No. 16 cells were serum-starved for 16 h and uninduced/induced for MEK5 expression by PonA for 0, 8, 16 and 24 h. Cells were stained by propidium iodide before analysis by flow cytometry. Duplicate samples were analysed for each of the time points. Bars represent mean values of the percentage of cells in G1, S or G2/M phase of the cell cycle. Experiments were repeated in triplicate. Left panel, uninduced; right panel, induced with PonA. MEK5 expression resulted in a two-fold increase in the percentage of cells in S phase. (**B**) Authenticity of clone. Clone EcRD No. 16 was either uninduced/induced with 5 *μ*M PonA for 24 h before screening whole cell lysates by immunoblotting with antibodies against HA tag (top), ERK5/phosphorylated ERK5 (middle) and *α*-tubulin (bottom). (**C**) Increased MEK5/ERK5 signalling was linked to increased Mcm2, geminin and Ki67 protein levels. EcRD No. 16 cells were either uninduced/induced with 5 *μ*M PonA for 24 h before screening whole cell lysates by immunoblotting with antibodies against Mcm2, geminin and Ki67.

**Figure 2:**
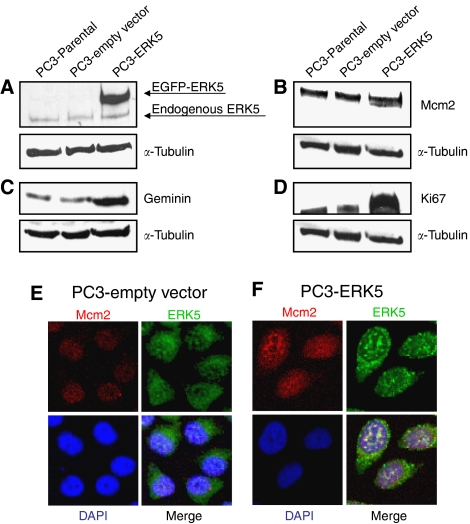
ERK5 overexpressing PC3 stable clone. (**A**) Immunoblots for ERK5 and *α*-tubulin of the parental PC3 line, the PC3 line transfected with empty vector, and the PC3-ERK5 stable clone. While the PC3 line has a background ERK5 level, the PC3-ERK5 stable clone expresses high levels of EGFP fused ERK5 protein. (**B**–**D**) Immunoblots of Mcm2, geminin and Ki67 in the parental PC3 line, PC3 line transfected with empty vector, and in the PC3-ERK5 stable clone. Levels of each protein are increased in the PC3-ERK5 stable clone. (**E**–**F**) Confocal fluorescence microscopy was used to investigate the expression profile and cellular localisation of Mcm2 in PC3 cells overexpressing ERK5 and those expressing the Empty Vector alone; cells were serum-starved, then stimulated with EGF. Mcm2 protein was detected in all cells with a predominantly nuclear localisation. Mcm2 expression was higher in PC3-ERK5 cells compared to PC3-Empty Vector cells. ERK5 expression was stronger in PC3-ERK5 cells compared to PC3-Empty Vector cells. DAPI was used to counterstain the nucleus.

To investigate potential linkages between the MEK5/ERK5 signalling pathway and the downstream DNA replication (or origin) licensing pathway (RLP), we first used an ecdysone-inducible system to express a constitutively activated mutant of MEK5 (MEK5D) in EcR293 cells (Mehta *et al*, 2003). Clone EcRD No. 16 showed negligible background MEK5 expression (Mehta *et al*, 2003). Treatment of cells for 24 h with Ponasterone A (PonA) resulted in a two-fold increase in the percentage of cells in S phase (Figure 1A). MEK5D expression was potently induced and was associated with a shift in the size of the ERK5 band, in keeping with ERK5 phosphorylation (Figure 1B). ERK5 is the only known substrate of MEK5 and, like other MAPK family members, it is activated by phosphorylation (Abe *et al*, 1996). Induction of the MEK5/ERK5 signalling pathway was coupled to a marked increase in the expression of Mcm2 and geminin, and also to upregulation of the standard proliferation marker Ki67 (Figure 1C). Next we used PC3 cells stably overexpressing ERK5 (PC3-ERK5 cells) to confirm our findings in a PCa model. PC3 cells were shown to have a background level of endogenous ERK5 (Figure 2A). The ERK5 overexpressing clone produced much higher levels of EGFP-fused ERK5 and WST-1 cell proliferation assays showed a ∼3-fold increase in proliferation compared to empty vector and parental controls. In keeping with the observations in the inducible system, the ERK5 overexpressing PC3 line expressed higher levels of Mcm2, geminin and Ki67 protein (Figure 2B–D). Indirect immunofluorescence studies also showed increased Mcm2 expression in ERK5 overexpressing cells (Figure 2E–F), confirming the immunoblot data, which give a population mean, at the level of individual cells. Taken together, these data demonstrate linkage of the MEK5/ERK5 signalling pathway with the replication licensing system in human prostatic cancer cells.

### Patient characteristics

To investigate coupling of MEK5/ERK5 signalling to the RLP *in vivo* and cell cycle kinetics of PCa, we analysed biopsy material from a cohort of PCa patients diagnosed after transurethral resection of the prostate (for clinical characteristics of the study cohort see Materials and Methods). Clinicopathological and immunohistochemical data were available for the entire cohort; however, five patients have been excluded from the survival analysis because survival times were unknown.

### DNA replication licensing in normal prostate and prostate cancer

The pattern of RLF expression was first assessed in morphologically normal prostate tissue present in the biopsy material. Expression of Mcm2, geminin and Ki67 was extremely low (<2%), consistent with our previous finding that loss of proliferative capacity which accompanies differentiation is coupled to repression of origin licensing through down-regulation of the Mcm2–7 RLFs (Stoeber *et al*, 2001; Eward *et al*, 2004). In PCa, on the contrary, Mcm2 and geminin protein levels were high, indicative of cell cycle re-entry (Kingsbury *et al*, 2005).

### Relationship between Mcm2, geminin and Ki67 protein expression and clinicopathological characteristics

**Figure 3:**
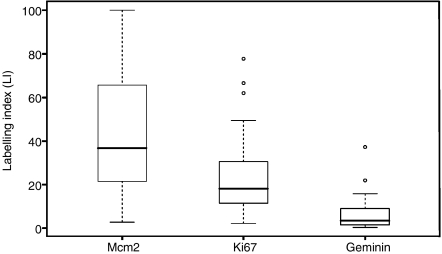
Relationships between marker expression in PCa. The median (solid black line), interquartile range (boxed), and range (enclosed by lines) of Mcm2, Ki67 and geminin expression are shown (outlying cases are shown by isolated points). The broader distribution of Mcm2 compared with Ki67 reflects the additional replication licensed but non-proliferating growth fraction, identified by Mcm2 but not Ki67. The low growth fraction identified by geminin compared with Ki67 is in keeping with its expression being restricted to S-G2-M phase. Overall, Mcm2 is expressed at significantly greater levels compared with Ki67, which itself is expressed more greatly than geminin (Wilcoxon signed-rank test, *P*<0.0001 in each case).

**Figure 4:**
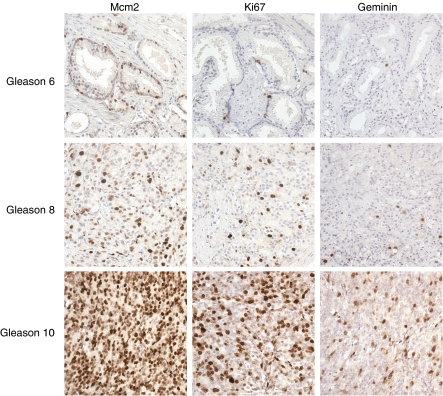
Photomicrographs of paraffin-embedded tissue sections of different PCa Gleason grades immunohistochemically stained with antibodies to Mcm2, Ki67 and geminin protein (positive cells stain brown). Protein expression is increased in higher grade tumours for each biomarker. Original magnification, × 400.

**Figure 5:**
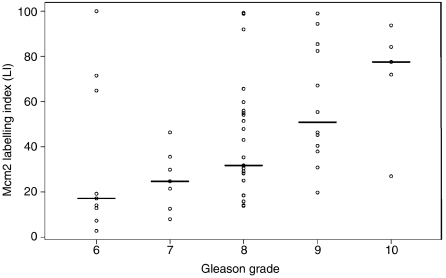
The median (solid black line) and range of Mcm2 expression are shown according to Gleason grade. Although there is an increase in median Mcm2 expression with increasing grade (*P*=0.0002, see also Table 1), a high Mcm2_LI_ does not preclude the possibility of low Gleason grade.

Distribution of LIs, median and inter-quartile ranges for each marker are shown in Table 1 and Figure 3, and immunohistochemical staining of representative cases of Gleason grade 6, 8 and 10 PCa is illustrated in Figure 4. Mcm2 protein expression in these tumours was significantly greater than Ki67 expression, which was itself significantly higher than geminin expression [median: Mcm2, 36.7% Ki67, 18.1% geminin, 3.4% (*P*<0.0001, Wilcoxon signed-rank test)]. Furthermore Mcm2 expression was distributed over a far broader range than Ki67 or geminin (Mcm2: 2.7–100% Ki67: 2.2–77.8% geminin: 0.3–37.2%). The high percentage of Mcm2-expressing cells compared to Ki67 can be explained by the presence of replication licensed but non-cycling cells in addition to proliferating cells (Stoeber *et al*, 2001; Blow and Hodgson, 2002). The low growth fraction identified by geminin compared with Ki67 is due to geminin expression being restricted to S, G2 and M phase (Eward *et al*, 2004; Wharton *et al*, 2004). There was a clear association of increasing Mcm2, geminin and Ki67 LIs with increasing Gleason grade (Mcm2, *P*=0.0002; geminin, *P*=0.0003; Ki67, *P*=0.004; Table 1 and Figure 4). Figure 5 shows more clearly the mean and distribution of Mcm2 LIs for each grade. Although there is an increase in mean Mcm2_LI_ with increasing grade, notably a high Mcm2_LI_ does not preclude the possibility of low Gleason grade. A statistically significant link between increasing Gleason grade and Ki67_LI_-geminin_LI_ (*P*=0.001) was also observed, indicating an increase in the population of cells entering G1 phase in more poorly differentiated tumours. There was no significant association between T stage and bony metastases with Mcm2, geminin or Ki67 expression.

### Relationship between MEK5, ERK5, Mcm2, geminin and Ki67 protein levels

We sought to identify the relationship between the MEK5/ERK5 growth signalling pathway and the RLFs Mcm2 and geminin, as well as the standard proliferation marker Ki67. Table 1 summarizes the links between these markers according to the degree of protein expression. The trends of increasing Mcm2, geminin and Ki67 expression with increased MEK5 expression are less marked. These weaker associations may reflect the fact that MEK5 lies upstream of ERK5 in the growth signalling pathway. We have previously stated that the RLP lies at a convergence point in growth signalling (Williams and Stoeber, 1999; Stoeber *et al*, 2001) and these data strongly support this hypothesis.

### Linkages between DNA replication licensing, MEK5/ERK5 signalling, and cell cycle kinetics in prostate cancer

**Figure 6:**
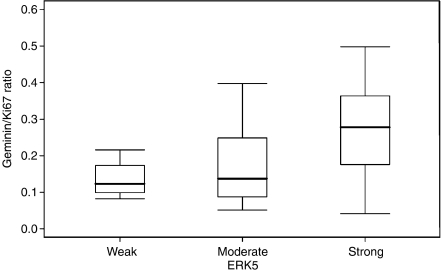
The median (solid black line), interquartile range (boxed), and range (enclosed by lines) of the geminin_LI_ to Ki67_LI_ ratio are shown according to ERK5 protein expression. There is a significant trend of increasing ERK5 expression with increasing geminin/Ki67 ratio (*P*=0.005; see also Table 1), indicative of a relative shortening in the length of G1 phase and thus accelerated cell cycle progression.

We have previously shown that RLF expression analysis can provide novel insights into the cell cycle kinetics of tumours. The Mcm2_LI_ minus Ki67_LI_ (Mcm2−Ki67) identifies the replication licensed but non-proliferating population of tumour cells, whereas the Ki67_LI_ minus geminin_LI_ (Ki67−geminin) represents the proportion of tumour cells in G1 phase (Stoeber *et al*, 2001, 2002; Dudderidge *et al*, 2005; Obermann *et al*, 2005). Geminin identifies the proportion of tumour cells traversing through S-G2-M phase, and therefore the geminin_LI_ to Ki67_LI_ ratio (geminin/Ki67) provides a measure of the relative length of G1 in dynamic cell populations, with higher geminin/Ki67 ratios corresponding to a shortening in the length of G1 phase (Eward *et al*, 2004; Wharton *et al*, 2004; Shetty *et al*, 2005). In this study, a statistically significant link between increasing Gleason grade and Ki67-geminin (*P*=0.001; Table 1) was observed, indicating an increase in the proportion of cells entering G1. Moreover, there was a significant relationship between increasing ERK5 protein expression and increasing Mcm2 (*P*=0.003) and Ki67 (*P*=0.009) expression (Table 1), recapitulating the data generated in the *in vitro* tissue culture model system (Figure 1). Furthermore, there was a statistically significant link between high ERK5 expression and increased Mcm2-Ki67 (*P*=0.01; Table 1), signifying an increase in the proportion of non-cycling tumour cells that are licensed for replication. We also observed a significant trend of increasing ERK5 expression with increasing geminin/Ki67 ratio, indicative of a relative shortening in the length of G1 phase and thus accelerated cell cycle progression (*P*=0.005; Table 1 and Figure 6).

### Mcm2, geminin and Ki67 expression and disease-free survival: univariate and multivariate analyses

**Figure 7:**
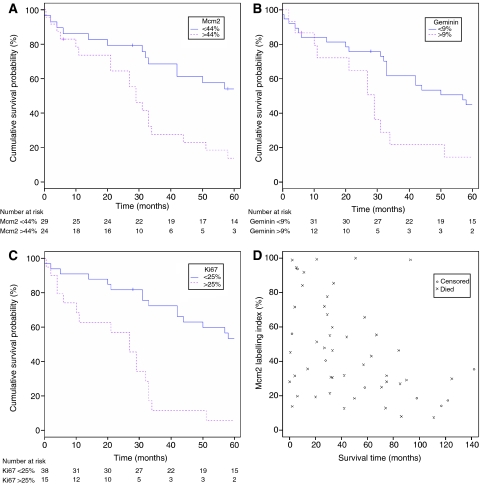
(**A**–**C**) Association between all-cause mortality and marker expression was assessed using Kaplan–Meier curves and the log-rank test, using the ‘minimum *P*-value approach’ with adjusted *P*-values and a significance level of 0.05. A statistically significant association was found between lower Mcm2 expression and increased survival time, and between lower Ki67 expression and increased survival time. After adjustment of the *P*-value, the trend between lower geminin expression and increased survival time was not statistically significant (details for each marker below). The number of patients at risk at each time-point for each group is shown below each graph. (**A**) Mcm2: groups defined by <44% (*n*=32), >44% (*n*=26), adjusted *P*-value=0.03. (**B**) Geminin: groups defined by <9% (*n*=43), >9% (*n*=15), adjusted *P*-value=0.22. (**C**) Ki67: groups defined by <25% (*n*=38), >25% (*n*=20), adjusted *P*-value=0.002. (**D**) Relationship between Mcm2_LI_ and survival time.

The association between all-cause mortality and biomarker expression was assessed using Kaplan–Meier curves and the log-rank test, using the ‘minimum *P*-value approach’ with adjusted *P*-values and a significance level of 0.05 (Faraggi and Simon, 1996). Survival analyses were performed for Mcm2, geminin and Ki67. A statistically significant association was found between lower Mcm2 protein expression and increased survival time (*P*=0.03; Figure 7A), and between lower Ki67 expression and increased survival time (*P*=0.002; Figure 7B). After adjustment of the *P*-value (Faraggi and Simon, 1996), the trend between lower geminin expression and increased survival time was not statistically significant (*P*=0.22; Figure 7C). The optimal cut points used were 44% for Mcm2, 25% for Ki67 and 9% for geminin. A Cox proportional hazards model was fitted to investigate trends between biomarker expression, the standard prognostic variables of Gleason grade, stage and survival. Increased expression of Mcm2 (*P*=0.005), geminin (*P*=0.04) and Ki67 (*P*=0.04) and the replication licensed but non-cycling cell population (Mcm2-Ki67; *P*=0.01) were all associated with significantly reduced survival time. However, only the strongest predictor of survival, Mcm2, was retained in the final model, as after adjustment for Mcm2 expression neither geminin, Ki67, Mcm2-Ki67, Ki67-geminin, geminin/Ki67 nor any of the other prognostic variables were significantly associated with survival time (hazard ratio for Mcm2=4.22; 95% CI (1.56, 11.45); *P*=0.005). Figure 7D shows the relationship between Mcm2 protein expression and survival. No significant relationships between Gleason grade and survival or stage and survival were identified. The presence of bony metastases was weakly associated with a reduction in survival time (hazard ratio 0.52; 95% CI (0.27, 1.00); *P*=0.05).

## DISCUSSION

Initiation of chromosomal replication is a critical step in growth control and thus important in tumourigenesis. It lies at the point of convergence of all oncogenic and transduction signalling pathways that trigger proliferation, making it a potentially attractive target for both diagnostic and therapeutic interventions (Williams and Stoeber, 1999). RLFs, which form the core of the initiation machinery, act as relay stations coupling growth regulatory and DNA damage response pathways to chromosomal replication. We and others have demonstrated that dysregulation of the replication initiation machinery is an early event in tumourigenesis and that the Mcm2-7 RLFs are powerful diagnostic and prognostic markers in a wide range of tumour types (Williams *et al*, 1998, 2004; Stoeber *et al*, 1999; Wharton *et al*, 2001, 2004; Davies *et al*, 2002; Going *et al*, 2002; Stoeber *et al*, 2002; Gonzalez *et al*, 2003; Kruger *et al*, 2003; Padmanabhan *et al*, 2004; Dudderidge *et al*, 2005; Korkolopoulou *et al*, 2005; Shetty *et al*, 2005). Recent reports have shown that the MEK5/ERK5 pathway is implicated in the regulation of cell proliferation, and that increased MEK5/ERK5 signalling results in increased cell proliferation, invasion and metastatic spread during prostate carcinogenesis (Kato *et al*, 1998; Mehta *et al*, 2003). In biochemical terms the MEK5/ERK5 pathway is uniquely specific in that MEK5 appears to function entirely through activation of ERK5, suggesting that this pathway could be an attractive target for anticancer therapy in PCa. Here we have investigated the effects of MEK5/ERK5 signalling on the DNA replication initiation machinery in prostate carcinogenesis to determine how this might influence origin licensing and cell cycle kinetics of this tumour type *in vivo*.

Repression of origin licensing is a powerful downstream mechanism by which human cells lower their proliferative capacity (Stoeber *et al*, 2001; Blow and Hodgson, 2002; Eward *et al*, 2004). Withdrawal of cells into reversible arrest (G0) or terminally differentiated states is coupled to tight downregulation of Mcm2–7 and the origin licensing inhibitor geminin (Stoeber *et al*, 2001; Eward *et al*, 2004; Kingsbury *et al*, 2005). However, MCM labelling identifies in addition to cycling cells, non-cycling cells with proliferative potential also. These MCM but not Ki67-expressing cells can be found, for example, in resting tissues such as pre-menopausal breast or primary oocytes, which retain proliferative capacity and can rapidly respond to growth stimuli (Stoeber *et al*, 2001; Eward *et al*, 2004). Their failure to initiate DNA synthesis and progress into S-G2-M phase is further reflected by lack of geminin expression (Shetty *et al*, 2005). Expression profiling of normal prostate shows only occasional basal Ki67, Mcm2, and geminin-positive cells, in keeping with repression of origin licensing during engagement of the differentiation programme in human tissues (Stoeber *et al*, 2001; Eward *et al*, 2004). In contrast, PCa shows high expression of RLFs and Ki67. The largest growth fraction is identified by Mcm2, reflecting identification of both cycling and non-cycling cells with proliferative potential. The lower growth fraction identified by geminin compared with Ki67 is in keeping with its expression being restricted to S-G2-M phase (Eward *et al*, 2004; Wharton *et al*, 2004; Dudderidge *et al*, 2005; Shetty *et al*, 2005). *In vitro*, increased MEK5/ERK5 signalling leads to increased Mcm2, geminin, and Ki67 protein levels. In PCa, increased MEK5/ERK5 signalling is coupled with an increase in the growth fraction identified by Ki67 and Mcm2 expression and in the number of replication licensed cells with proliferative potential (Mcm2_LI_-Ki67_LI_). Interestingly, increased MEK5/ERK5 signalling is also linked to an increase in the geminin_LI_ to Ki67_LI_ ratio. Assuming that the tumour cells are not significantly delayed in their progression through S-G2-M phase by DNA damage checkpoints, an increase in the geminin_LI_ to Ki67_LI_ ratio is indicative of a relative shortening in the length of G1 phase, the ratio tending towards unity (∼1) as G1 phase reduces in length (Wharton *et al*, 2004; Eward *et al*, 2004; Obermann *et al*, 2005; Shetty *et al*, 2005). Thus increased MEK5/ERK5 signalling appears to be coupled to accelerated cell cycle progression in PCa. Moreover, the observed changes in cell cycle parameters are indicative of increased proliferative capacity in response to MEK5/ERK5 signalling.

Previous studies exploiting *in vitro* and *in vivo* model systems have shown that engagement of the somatic differentiation programme in human cells is coupled to downregulation of the Mcm2–7 and geminin RLFs, as cells exit the proliferative cycle and enter the terminally differentiated state (Williams *et al*, 1998; Stoeber *et al*, 2001; Eward *et al*, 2004). In this study the block to the differentiation programme in PCa, indicated by increasing Gleason grade, is associated with increased expression of Ki67, Mcm2 and geminin proteins. We also observed an increase in the number of cells entering G1 phase with increasing Gleason grade, indicated by an increase in Ki67_LI_-geminin_LI_. Importantly while a clear relationship between Mcm2 protein expression and Gleason grade has been shown in this study, high Mcm2 expression does not preclude the possibility of low Gleason grade, indicating a broad spectrum of growth potential within each tumour grade. Interestingly, there is no association with T stage or bony metastases and Mcm2, geminin or Ki67 protein expression. Increased expression of MCM RLFs in response to arrested differentiation (maturation arrest) has also been observed in other tumour types (Williams *et al*, 1998; Going *et al*, 2002; Stoeber *et al*, 2002) and is currently being assessed as a cancer screening tool in multi-centre trials (National Cancer Research Network Trial ID 1279). This finding is in keeping with our previous analysis of radical prostatectomy specimens, in which we showed that Mcm2 expression, although showing an association with primary grade and Gleason score, did not show statistical association with T stage or metastasis (Meng *et al*, 2001). This may be a reflection of the fact that PCa metastasis is not as dependant on abrogation of cell proliferation control as compared with other factors such as loss of cell–cell/cell–extracellular matrix adhesion, increased cell motility, basement membrane penetration, angioneogenesis, and immune escape through impaired expression of surface tumour antigens (Hrouda and Kirby, 1998).

Although increased Mcm2, geminin and Ki67 protein expression and the replication licensed but non-cycling cell population (Mcm2_LI_-Ki67_LI_) were all associated with significantly reduced survival time, only Mcm2 was identified as an independent predictor of survival on multivariate analysis. We have previously observed that the Mcm2_LI_ provides information with respect to disease-free survival in PCa patients undergoing radical prostatectomy (Meng *et al*, 2001), and our findings are also in keeping with the recent report that Mcm7 amplification and overexpression is associated with worse tumour grade and relapse (Ren *et al*, 2006).

In summary, analysis of the origin licensing pathway in PCa has shown that dysregulation of the DNA replication licensing machinery is a major event during prostate carcinogenesis. The broad distribution of Mcm2 protein expression within Gleason grades and the fact that this biomarker is an independent predictor of survival indicate that MCM RLFs are important as potential stratification tools for identification of PCa with aggressive phenotypes. Moreover, our data show that induction of MEK5/ERK5 signalling is linked to activation of the DNA replication licensing pathway in PCa, which is associated with marked changes in cell cycle kinetics of these tumours indicative of a shift to increased growth potential. The strong prognostic value of MCM RLFs appears to arise as a result of their function as relay stations coupling upstream growth regulatory pathways to genome duplication. Recently, the DNA replication licensing pathway has been proposed as a selective target for anti-cancer therapy, since tumour cells appear to be deficient in a putative licensing checkpoint. Thus anti-cancer therapy directed at both MEK5/ERK5 signalling and the DNA replication licensing machinery offers an attractive approach for multitargeted therapy in PCa.

## Figures and Tables

**Table 1:** Relationship between Mcm2 _LI_, geminin _LI_, Ki67 _LI_, Mcm2 _LI_ -Ki67 _LI_, Ki67 _LI_ -geminin _LI_ and geminin _LI_ /Ki67 _LI_ with other factors^a^

		**n**	**Mcm2**	**Geminin**	**Ki67**	**Mcm2-Ki67**	**Ki67-geminin**	**Geminin/Ki67**
Gleason grade	6	9	17 (10–68)	1.5 (0.9–9.6)	13 (9.7–23)	6.7 (0–36)	11 (6.3–17)	13 (8.5–31)
	7	7	24 (13–36)	1.7 (1.4–2.9)	15 (4.8–24)	7.7 (5.3–11)	12 (4.1–21)	13 (11–20)
	8	25	32 (27–55)	4.3 (2.2–9.5)	17 (9.3–28)	19 (10–35)	12 (5.5–17)	28 (16–41)
	9	12	51 (39–85)	4.6 (1.6–9.6)	30 (19–38)	30 (5.6–45)	23 (15–32)	19 (11–26)
	10	5	78 (49–89)	15 (5.5–30)	49 (26–72)	16 (4.2–42)	34 (21–43)	32 (20–40)
*P*-value^b^			0.0002	0.0003	0.004	0.06	0.001	0.21
Stage	1	13	32 (16–60)	2.7 (0.9–8.7)	23 (9.8–26)	9.9 (3.5–48)	17 (5.4–22)	12 (8.7–30)
	2	15	43 (25–92)	2.9 (1.9–11)	15 (12–28)	30 (18–62)	12 (9.1–17)	20 (15–39)
	3	17	38 (24–75)	3.7 (1.9–11)	17 (9.8–34)	16 (7.8–32)	13 (5.5–23)	25 (15–36)
	4	5	48 (32–69)	10 (5.1–13)	37 (20–46)	16 (6.4–27)	27 (11–36)	23 (20–38)
*P*-value^b^			0.49	0.23	0.07	0.77	0.29	0.08
Bone metastases	Absent	28	46 (29–76)	3.7 (2.1–11)	22 (13–34)	19 (8.9–45)	16 (11–27)	23 (12–35)
	Present	28	29 (14–63)	2.5 (1.2–8.9)	15 (8.9–24)	10 (3.5–29)	12 (5.6–19)	17 (12–32)
*P*-value^c^			0.02	0.11	0.17	0.12	0.12	0.53
ERK5	Weak	4	12 (3.9–58)	1.2 (0.6–8.1)	9.7 (6.5–39)	2.9 (0–20)	8.5 (5.9–30)	12 (9.1–20)
	Moderate	18	27 (14–43)	2.2 (1.3–5.5)	17 (12–25)	9.0 (3.1–26)	13 (9.5–20)	14 (8.7–26)
	Strong	36	47 (30–71)	5.3 (2.4–11)	23 (12–35)	21 (11–35)	16 (9.0–26)	28 (17–37)
*P*-value^b^			0.003	0.07	0.009	0.01	0.27	0.005
MEK5	Weak	6	22 (17–96)	1.8 (1.2–9.5)	12 (7.1–20)	8.9 (5.7–76)	8.4 (3.7–16)	19 (10–49)
	Moderate	25	38 (23–58)	2.7 (1.1–6.5)	20 (12–32)	17 (2.5–31)	16 (10–26)	16 (8.6–23)
	Strong	26	44 (26–73)	7.5 (2.2–11)	21 (13–33)	18 (7.7–36)	15 (8.5–24)	30 (16–35)
*P*-value^b^			0.38	0.17	0.03	0.5	0.41	0.02
Overall	58		37 (21–66)	3.4 (1.5–9.3)	18 (11–31)	17 (6.3–33)	15 (8.5–22)	19 (12–34)

Note: Use significance level of 0.01 to account for multiple testing.

Abbreviations: ERK5=extracellular signal-regulated kinase-5; geminin_LI_/Ki67_LI_=measure of the relative length of G1 phase; Ki67_LI_-geminin_LI_=proportion of tumour cells in G1 phase; LI=labelling index; Mcm2_LI_-Ki67_LI_=replication licensed but non-cycling population of tumour cells; MEK5=mitogen/extracellular-signal-regulated kinase kinase 5.

a Values shown are median (inter-quartile range), expressed as percentages.

b Jonckheere–Terpstra test.

c Mann–Whitney *U*-test.
